# The Potential Protective Effect of Estrogen: A Plausible Theory for why COVID-19 Mortality is Lower in Females

**DOI:** 10.1016/j.ijwd.2020.04.011

**Published:** 2020-04-30

**Authors:** Dedee F. Murrell, Jenny E. Murase

**Affiliations:** a Department of Dermatology, St. George Hospital, UNSW Faculty of Medicine, Sydney, Australia; b Department of Dermatology, UCSF, San Francisco, CA, United States

**Keywords:** COVID-19

The current COVID-19 pandemic is upon us, and factors that contribute to the mortality in apparently healthy people are not yet understood. Epidemiologic statistics from the source at Wuhan and death rates in Iran, Italy, and Spain reveal almost double the mortality rates in men compared with women (Guan et al., 2020, Li et al., 2020, Shi et al., 2020). Some excess deaths are attributed to the increased prevalence of smoking among men in China and the attendant comorbidities associated with chronic smoking; however, in the European Union, many women smoke as well, yet mortality is still much higher in men (Guan et al., 2020). Could estrogen play a role?

We know that the COVID-19 virus uses its spike protein to gain entry into cells via the angiotensin converting enzyme-2 (ACE-2) protein, a receptor for the virus that is expressed in a number of tissues in humans, including the mucosal linings of the heart, kidneys, lungs, nose, oral cavity, and eyes (Harmer et al., 2002). The expression of ACE-2 is reduced by estrogen and increased by testosterone (Komukai et al., 2010, White et al., 2019, Zapater et al., 2004). Hence, women, who have much lower levels of testosterone than men, receive a lower total viral load on infection unless they are postmenopausal and not on estrogen replacement therapy.

ACE-2 levels are low in newborns and increase with age, which is another factor that may protect the young from COVID-19 (Dalpiaz et al., 2015).

Ibuprofen and potentially other nonsteroidal anti-inflammatory drugs increase the levels of ACE-2 and may be taken by women when they have headaches or menstrual cramps or by men with sporting injuries. These may explain why some younger patients become vulnerable; these drugs are sold over the counter, and this information may not be available to doctors when sick patients arrive at the hospital.

ACE inhibitors and angiotensin II receptor blockers taken for hypertension and a variety of other cardiac and renal indications also increase ACE-2 tissue expression via a feedback loop. Recently published guidelines from various professional societies state that there is insufficient evidence to cease use of such medications during this pandemic. Nevertheless, large randomized controlled trials previously found no outcome differences in the complications of these drugs and calcium channel blockers in terms of strokes and cardiac deaths (Turnbull and Blood Pressure Lowering Treatment Trialists' Collaboration, 2003). Hence, for the period of the pandemic, would it not be worth considering this on a case-by-case basis, depending on the patients’ other risk factors, including male sex, smoking, immunosuppression, and other comorbidities? Many patients with severe psoriasis have all the aforementioned comorbidities and are taking these medications for hypertension.

Behavioral aspects could also reduce survival in men. Women are more likely to seek medical attention early in the course of an illness compared with men (Wang et al., 2013).

1. What advice can we glean from the above facts which may assist the population at large? Men should consider reducing their consumption of androgenic steroid supplements. Many protein powders taken by young and older men contain dehydroepiandrosterone, an androgenic steroid, and potentially other androgens
2. Postmenopausal women and women taking progestin-only contraceptives may wish to speak with their treating physicians about temporarily using estrogen-containing alternatives, if there are no contraindications.
3. Transgender individuals may wish to reduce testosterone supplements or take testosterone blockers, depending on the situation, to reduce testosterone exposure.
4. Avoid taking ibuprofen and other nonsteroidal antiinflammatory drugs if possible, and take acetaminophen for pain instead.
5. If taking ACE inhibitors or angiotensin II receptor blockers for hypertension, consider switching to another agent during the pandemic.

## Conflict of Interest

None.

## Funding

None.

## Study approval

The author(s) confirm that any aspect of the work covered in this manuscript that has involved human patients has been conducted with the ethical approval of all relevant bodies.
